# Predictors of long term outcomes of IgA nephropathy

**DOI:** 10.1186/1753-6561-6-S4-P50

**Published:** 2012-07-09

**Authors:** U Kabir, T Rahman, D Connaughton, P Conlon

**Affiliations:** 1 Royal College of Surgeons in Ireland, Ireland

## Introduction

IgA Nephropathy (IgAN) was first described by Berger and Hinglais in 1968. The correlations between clinical as well as histopathological features on presentation and disease progression still remain unclear.

## Aim

To identify the key predictors of long term renal outcomes in IgAN.

## Methods

Two hundred patients with biopsy proven IgAN were selected and their clinical as well as histopathological data was analysed for progression to End-stage renal disease (ESRD).

## Results

98 patients were used as the selected cohort. 21.5% of patients developed ESRD within 5 years and 28.8% within 10 years. Univariate studies showed that interstitial fibrosis, glomerulosclerosis, presence of fibrocellular crescents, proteinuria (g/24hrs) and raised serum creatinine levels at the time of biopsy were significant. (P value of <0.001 and 95% CI considered significant.) In the multifactorial analysis interstitial fibrosis and raised serum creatinine, lost their significance. (P value of <0.05 and 95% CI considered significant.)

## Conclusions

Combination of proteinuria, glomerulosclerosis and fibrocellular crescents may contribute to poor renal outcomes in patients with IgAN. Interstitial fibrosis and raised serum creatinine levels may have solitary effects on disease progression as well.

